# Predicting Prognosis of Early-Stage Mycosis Fungoides with Utilization of Machine Learning

**DOI:** 10.3390/life14111371

**Published:** 2024-10-25

**Authors:** Banu İsmail Mendi, Hatice Şanlı, Mert Akın Insel, Beliz Bayındır Aydemir, Mehmet Fatih Atak

**Affiliations:** 1 Department of Dermatology, Niğde Ömer Halisdemir University Training and Research Hospital, Niğde 51000, Türkiye; 2 Department of Dermatology, Faculty of Medicine, Ankara University, Ankara 06620, Türkiye; erdi@medicine.ankara.edu.tr (H.Ş.); bbayindir@ankara.edu.tr (B.B.A.); 3 Department of Chemical Engineering, Yıldız Technical University, İstanbul 34220, Türkiye; makinsel@yildiz.edu.tr; 4 Department of Dermatology, New York Medical College, Valhalla, NY 10595, USA; fatih9164@hotmail.com

**Keywords:** machine learning, mycosis fungoides, prognosis

## Abstract

Mycosis fungoides (MF) is the most prevalent type of cutaneous T cell lymphomas. Studies on the prognosis of MF are limited, and no research exists on the potential of artificial intelligence to predict MF prognosis. This study aimed to compare the predictive capabilities of various machine learning (ML) algorithms in predicting progression, treatment response, and relapse and to assess their predictive power against that of the Cox proportional hazards (CPH) model in patients with early-stage MF. The data of patients aged 18 years and over who were diagnosed with early-stage MF at Ankara University Faculty of Medicine Hospital from 2006 to 2024 were retrospectively reviewed. ML algorithms were utilized to predict complete response, relapse, and disease progression using patient data. Of the 185 patients, 94 (50.8%) were female, and 91 (49.2%) were male. Complete response was observed in 114 patients (61.6%), while relapse and progression occurred in 69 (37.3%) and 54 (29.2%) patients, respectively. For predicting progression, the Support Vector Machine (SVM) algorithm demonstrated the highest success rate, with an accuracy of 75%, outperforming the CPH model (C-index: 0.652 for SVM vs. 0.501 for CPH). The most successful model for predicting complete response was the Ensemble model, with an accuracy of 68.89%, surpassing the CPH model (C-index: 0.662 for the Ensemble model vs. 0.543 for CPH). For predicting relapse, the decision tree classifier showed the highest performance, with an accuracy of 78.17%, outperforming the CPH model (C-index: 0.782 for the decision tree classifier vs. 0.505 for CPH). The results suggest that ML algorithms may be useful in predicting prognosis in early-stage MF patients.

## 1. Introduction

Mycosis fungoides (MF) is the most prevalent type of cutaneous T cell lymphoma (CTCL) with an incidence rate of 4.1/1,000,000 person–years, and the majority consists of early-stage MF patients [1,2,3,4]. Despite the vast majority of MF patients remaining in the early stages for long periods, approximately 20–25% of patients eventually progress to advanced stages, which can be fatal. Hence, the early detection of patients at risk of progression to advanced stages is critical [5,6]. Various factors associated with progression have been identified in the literature, including male gender, advanced age, clinical stage, elevated levels of lactate dehydrogenase, blood eosinophilia, folliculotropism, and large-cell transformation [5,7,8,9]. Benton et al. developed the Cutaneous Lymphoma International Prognostic Index (CLIPI) based on several of these factors to predict prognosis in patients with MF [9]. However, there have been ongoing controversies regarding the generalizability and validation of CLIPI in other cohorts [10,11]. Nevertheless, predicting patients with a higher risk of progression in MF patients still poses a significant challenge.

The management of early-stage MF is determined primarily by the stage of the disease. However, there is no specific algorithm for the treatment of early-stage disease, and individualized treatments are required according to the patient’s needs, side effect profiles, and likelihood of relapse. The treatment procedure may be prolonged in patients who are resistant to treatment or experience a relapse, possibly resulting in decreased patient compliance to the treatment and decreased quality of life. Therefore, it is crucial to identify these patients prior to treatment and establish an appropriate treatment regimen [12,13,14].

The interpretive capabilities of artificial intelligence (AI) and machine learning (ML) techniques have shown potential in predicting disease progression, determining appropriate treatments, and guiding follow-up strategies. While numerous studies have explored the use of AI for diagnosing various skin diseases, research specifically focusing on the application of AI for MF remains limited [15,16]. For instance, Thomsen et al. employed deep learning (DL), a subset of ML, to distinguish CTCL from eczema by analyzing skin images. Their binary classification model, VCG-16, achieved an accuracy of 81.46% [17]. Another study utilized a deep convolutional network, the single-shot multibox detector, for diagnosing MF, atopic dermatitis, and psoriasis, achieving an overall accuracy of 93% in multi-class classification. When applied specifically to MF diagnosis, the sensitivity and accuracy rates were 94% and 98%, respectively [18]. Furthermore, Karabulut et al. developed a DL model capable of facilitating MF diagnosis using hematoxylin–eosin-stained micrographs. Initially, nuclei were detected, and their properties were extracted. Subsequently, a multi-layer perceptron classifier was employed to identify lymphocytes among the detected nuclei. Finally, the Random Forest classifier method was utilized to distinguish between MF and non-MF lymphocytes. The algorithm demonstrated an average predictive accuracy of 94.2% [19]. Despite these advancements in AI applications for the diagnosis of MF, to the best of our knowledge, there has been no study evaluating AI in predicting the prognosis of MF in the literature to date. Our study aims to fill this critical gap by evaluating and comparing the predictive performance of various ML algorithms in determining prognosis, including disease progression, treatment response, and relapse in early-stage MF patients.

## 2. Materials and Methods

The data of 185 patients aged 18 years and older, who were admitted to Ankara University Faculty of Medicine Hospital between January 2006 and February 2024 and diagnosed histopathologically and immunohistochemically with MF and determined to be in the early stage according to the International Society of Cutaneous Lymphomas (ISCL) criteria [2], were retrospectively reviewed. Institutional Review Board approval was obtained, and informed consent forms were waived due to the retrospective nature of the study. Gender, age at diagnosis, age of onset of lesions, time to diagnosis, morphological, histopathological, immunohistochemical, laboratory findings, Tumor–Node–Metastasis–Blood (TNMB) stage at diagnosis, and initial treatments were recorded. Response to initial treatment and relapse were recorded according to ISCL clinical endpoints and response criteria. Complete response was defined as 100% clearance of skin lesions, partial response as 50% to 99% clearance of skin disease from baseline without tumors, stable disease as less than 25% increase in skin disease from baseline to less than 50% clearance without tumors, and progressive as greater than 25% increase in skin disease from baseline or development of tumors or loss of response (in those with a complete or partial response and nadir plus skin score increase greater than the sum of the 50% baseline score). Any recurrence of disease in complete responders was defined as relapse [13]. Response to initial treatment was evaluated as complete response/no complete response during training and testing of the models.

The ability of the CLIPI index [9] to predict progression within our cohort was assessed in this study. The CLIPI index encompasses distinct criteria for early and advanced stages, with each criterion assigned 1 point. The early-stage criteria encompass male gender, age over 60 years, plaque lesion, folliculotropism, and N1/Nx status. As per the index, 0–1 points indicate low risk, 2 points suggest medium risk, and 3–5 points indicate high risk. In our study, patients were also stratified into groups based on the CLIPI. The significance of CLIPI groups in predicting progression risk, Concordance index (C-index), and Area Under the ROC Curve (AUC) values were subsequently determined. Additionally, prognostic analyses for complete response, relapse, and progression were conducted utilizing the Cox proportional hazards model. Statistical significance was set at *p* < 0.05. C-index and AUC values were calculated for both the multivariate Cox proportional hazards models and the CLIPI score. Data were analyzed on XLSTAT version 2024.2.2 (statistical analysis software) at a 95% confidence level.

Several ML algorithms are utilized in this study to predict complete response, relapse, and progression. These algorithms include decision trees [20], discriminant classifiers [21], logistic regression [22], Naive Bayes classifiers [23], Support Vector Machines (SVMs) [23], k-nearest neighbors (KNNs) [24], ensemble methods [25], and shallow neural networks [26]. Deep learning algorithms are left out of the scope of this study since these algorithms require a large number of data to perform well [27] and may result in overfitting with limited data, which reduces the model’s generalization capability [28].

The Bayesian optimization [29] algorithm was employed to identify the optimal hyperparameters for the artificial neural network (ANN) architecture, particularly in scenarios where the computational complexity is high and the objective function is not known. Bayesian optimization was chosen for its exceptional performance in such situations [30]. It uses a surrogate probabilistic model, often a Gaussian process, to approximate the objective function based on past observations. An acquisition function guides the selection of the next point to evaluate by balancing the trade-off between the exploration of uncertain regions and the exploitation of areas with known high performance. The acquisition function of “expected improvement per second plus” was utilized for this study with a maximum of 30 iterations being allowed for each type of ML model.

The effectiveness of ML models can be assessed in various ways. A way to test classification accuracy is the confusion matrix, where true positive (TP), true negative (TN), false positive (FP), and false negative (FN) are specified as the principal component of the matrix. Some of the performance metrics that can be acquired using the confusion matrix are correct classification (accuracy), true positive rate (TPR), and false negative rate (FNR). The *F*1 Score is also a crucial metric for evaluating the performance of a classification, evaluated in Equation (1) as follows [31]:

(1) $$F1\;Score=\frac{2\cdot Precision\cdot Recall}{Precision+Recall}$$

where $Precision=\frac{TP}{TP+FP}$ is known as the positive predictive value, and $Recall=\frac{TP}{TP+FN}$ is also referred as the sensitivity.

**Figure 1:**
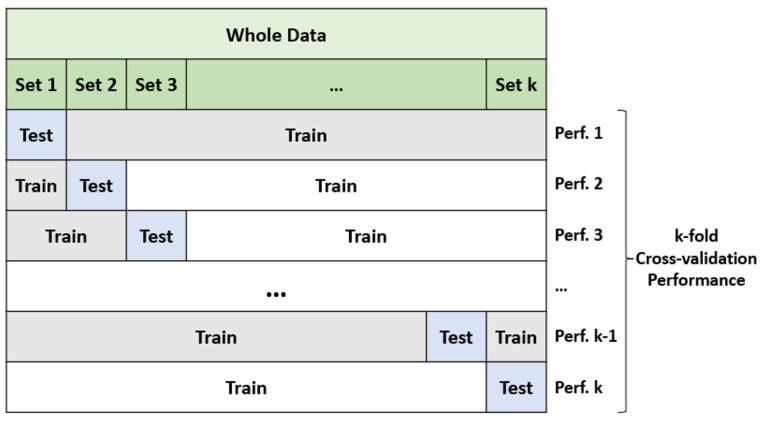
Fundamentals of k-fold cross-validation.

**Figure 2:**
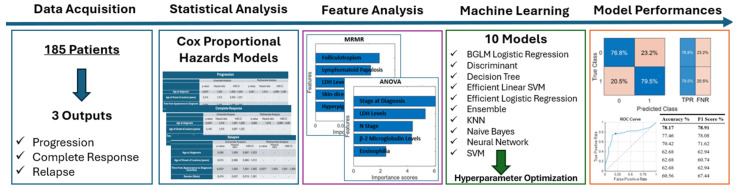
Flowchart of the study of lactate dehydrogenase (LDH): Bayesian Generalized Linear Model (BGLM); Support Vector Machine (SVM); k-nearest neighbor (KNN); true positive rate (TPR); false negative rate (FNR); receiver operating characteristic (ROC).

Typically, ML algorithms employ two sorts of model performance evaluation methodologies. Two often-used assessment methods are hold-out validation and k-fold cross-validation. The hold-out assessment approach partitions the complete dataset into three distinct groupings. The sets consist of a validation set, a training set, and a hold-out/test set. The model is trained using the training set, and its validity is assessed using the validation set. The best parameters for the approach are then determined. The hold-out and test sets are utilized to assess the performance of the model. A classification algorithm is employed to evaluate the model. The hold-out approach [32] is a technique used for constructing a model. K-fold cross-validation is a widely used method in applications where data are divided into k subsets. The k-fold cross-validation [33] method is an evaluation technique that involves randomly partitioning a dataset into k equal-sized subsets. In k-fold cross-validation, subsets are used to build the model, while one subset is reserved for pattern validation. This indicates that there is a greater emphasis on constructing the model rather than on the validation approach. Nevertheless, certain research studies have confirmed that placing greater emphasis on the validation approach can result in an enhancement in model selection [32]. This evaluation model predicts the outcome that will be obtained in the dataset of the statistical study. Cross-validation allows for an impartial measurement of prediction error without the need to divide the data into separate training and testing groups. The cross-validation method’s fundamentals are demonstrated in Figure 1. In this study, the accuracy metric of all models is evaluated by the 10-fold cross-validation [33] method. The confusion matrix for the best-performing model for each observed output is also represented by utilizing the whole data, including TPR and FPR values. The flowchart of the study is summarized in Figure 2.

## 3. Results

**Figure 3:**
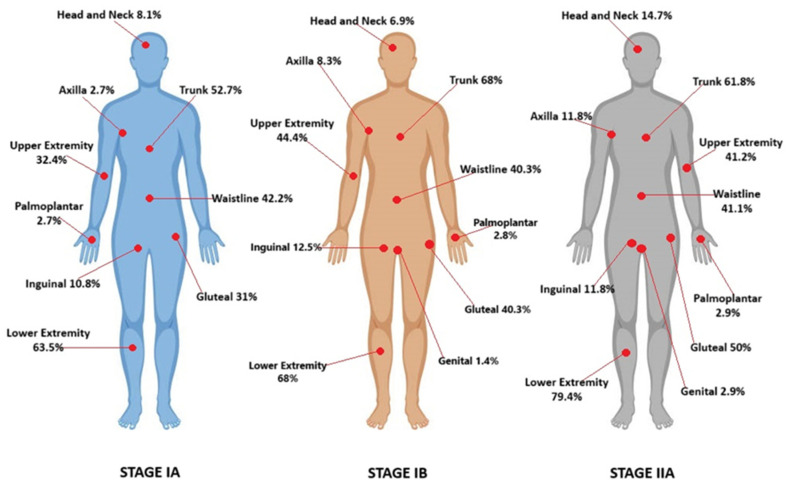
Affected regions according to stages.

Demographic and clinical characteristics of patients are summarized in Table 1. Here, all the data were categorical, except for age at diagnosis, age of onset lesions, follow-up time, and time from appearance to diagnosis. Of the 185 patients, 94 (50.8%) were female, and 91 (49.2%) were male, and the average age was 50.8 years. The mean follow-up time was 5.6 years, and the mean time from the onset of the lesions to diagnosis was 56.3 months. Seventy-six (41%) of the patients were stage IA, seventy-three (39.5%) were stage IB, and thirty-six (19.5%) were stage IIA. According to morphological features, one hundred and twenty (64.8%) patients were erythematous, thirty-five (18.9%) patients were hyperpigmented, thirteen (7%) patients were poikilodermic, nine (4.8%) patients were folliculotropic, seven (3.8%) patients displayed pigmented purpuric dermatosis-like (PPD-like) features, three (1.6%) patients were hypopigmented, one (0.5%) patient was of the ichthyosiform type, and one (0.5%) patient had Granulomatous Slack Skin (GSS). One hundred and eight (58.4%) patients reported pruritus. In terms of disease distribution regions, sixteen patients exhibited involvement in the head and neck region, seventy patients in the upper extremities, twelve patients in the axilla, one hundred and nine patients in the trunk, seventy-six patients in the waist area, sixty-nine patients in the gluteal region, twenty-one patients in the inguinal region, one hundred and twenty-three patients in the lower extremities, five patients in the palmoplantar area, and two patients in the genital region. Affected regions according to stages are shown in Figure 3.

Histopathologically, one hundred and nineteen (64.3%) patients were classical, twenty-eight (15.1%) were folliculotropic, five (2.7%) were PPD-like, one (0.5%) was GSS, one (0.5%) was syringotropic, one (0.5%) was granuloma annulare-like, and nine (4.8%) patients had Large Cell Transformation (LCT). In immunohistochemical examination, one hundred and fifty-three (82.7%) patients were CD4 (+) CD8 (−), twenty-eight (15.1%) patients were CD4 (−) CD8 (+), two (1%) patients were CD4 (+) CD8 (+), and two (1%) patients were CD4 (−) CD8 (−). CD30 (+) was detected in four (2.1%) patients.

According to the type of treatment, one hundred and forty-nine patients received skin-directed treatment, thirty patients received combined treatment, and six patients underwent systemic treatment. PUVA therapy was the most commonly used skin-directed treatment. The most frequently used initial treatment was PUVA therapy (41.6%), followed by Narrowband UVB (nbUVB, 35.1%).

Complete response to initial treatment was observed in 114 (61.6%) patients. Relapse occurred in 69 (37.3%) patients after initial treatment. There was progression in 54 (29.2%) patients during the follow-up period. Of the patients who progressed, seven patients progressed from stage IA to IB, nine patients from IA to IIA, six patients from IA to IIB, nine patients from IB to IIA, two patients from IB to IIB, nine patients from IB to IIIA, one patient from IIA to IIB, ten patients from IIA to IIIA, and one patient from IIA to IVA2. Patient characteristics according to progression, complete response, and relapse are shown in Table 2.

### 3.1. Cox Proportional Hazards Models

#### 3.1.1. Progression

Demographic, clinical, histopathological, and laboratory characteristics, treatment types, and their correlations with progression are depicted in Table 3. Based on the CPH model, factors such as age, pruritus, elevated LDH levels, and Nx pathology were identified as correlated with progression. Classic pathology, skin-directed treatment, and PUVA therapy were associated with a decreased risk of progression. Through multivariate analysis, high LDH levels were linked to progression, while classic pathology was associated with a reduced progression risk. The C-index for progression in the multivariate CPH model was determined to be 0.501.

##### CLIPI

The *p*-value for CLIPI regarding progression risk was 0.011 (*p* < 0.05), with a corresponding C-index of 0.278 (CPH model). The AUC value was at 0.655.

#### 3.1.2. Complete Response

Demographic, clinical, histopathological, and laboratory characteristics, treatment types, and their correlations with complete response are depicted in Table 4. In the univariate CPH analysis, factors such as age, gender, disease stage, beta-2-microglobulin levels, and initial treatment were linked to complete response. Male gender, stage IIA disease, elevated β-2 microglobulin levels, and NB-UVB treatment were associated with reduced complete response, whereas PUVA therapy correlated with improved complete responses. In the subsequent multivariate analysis, male gender, β-2 microglobulin levels, and PUVA treatment emerged as significant predictors of complete response. The C-index for complete response in the multivariate CPH model was 0.543.

#### 3.1.3. Relapse

Demographic, clinical, histopathological, and laboratory characteristics, treatment types, and their correlations with relapse are depicted in Table 5. In the univariate CPH model, variables, including the duration from lesion onset to diagnosis, DBUVB treatment, and PUVA therapy, were found to be associated with recurrence. A longer duration between lesion onset and diagnosis, as well as NBUVB treatment, were linked to an elevated risk of relapse, while PUVA treatment was correlated with a reduced risk of relapse. In the multivariate analysis, the time to diagnosis and PUVA treatment were specifically identified as factors associated with relapse. The C-index for complete response in the multivariate CPH model was 0.505.

### 3.2. Implementation of Machine Learning Models

#### 3.2.1. Data Preprocessing and Feature Importances

**Figure 4:**
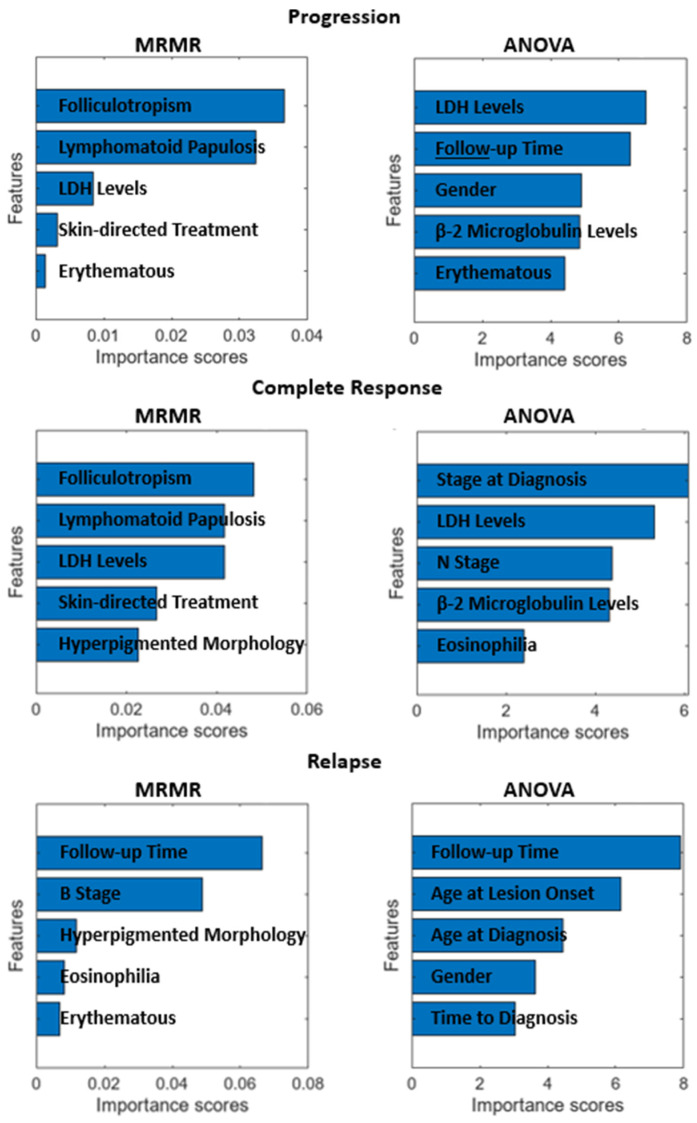
Feature importance scores for each response obtained by MRMR and ANOVA.

Before performing any kind of modeling study, it is best practice to analyze the data and consider data or feature elimination, augmentation, or cleaning. To this end, we have utilized MRMR and ANOVA feature scoring algorithms to deduce the most important features for progression, complete response, and relapse. The MRMR algorithm tends to select a subset of features having the most correlation with the output, while the ANOVA algorithm favors the selection of attributes characterized by minimal intra-class variance and maximal inter-class variance [34]. Figure 4 illustrates the ten most important features, in order, for each output and algorithm. Since there were no significantly prevalent estimators for progression, complete response, and relapse, all the features are kept as is for the obtainment of ML models in the next section. However, there were five patients with null data for several features, and thus, these data were eliminated whilst modeling. The elimination of these data does not affect the statistics given in Table 1 significantly since the presence of null data was random, and the eliminated data are of low proportion when compared to the whole dataset.

#### 3.2.2. Progression

**Figure 5:**
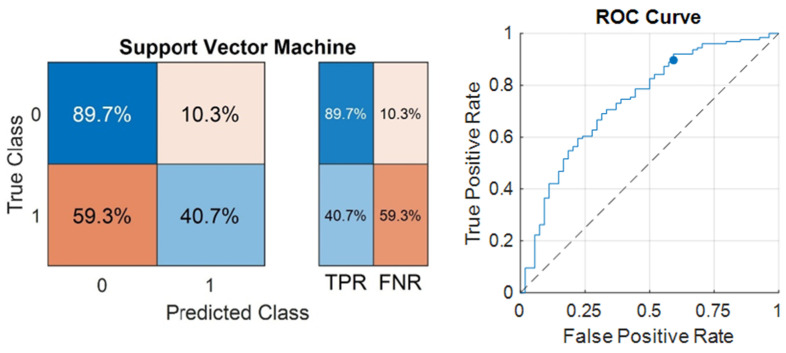
Confusion matrix and ROC Curve of the best-performing model in the estimation of progression.

The most successful model in predicting progression was SVM, with an accuracy of 75.00% and an F1 Score of 49.44% (given in Table 6). The sensitivity (TPR) of the model was 40.7%, and the specificity (TNR) was 89.7% (Figure 5). The AUC of the model was 0.73927. The ROC Curve for the SVM was also illustrated in Figure 2. The optimum hyperparameters were also obtained by utilizing the Bayesian optimization for the SVM model. Accordingly, the optimum kernel function was determined as linear with a kernel scale of 1, the optimum box constraint level was 4.8087, and the optimum multiclass method was one-vs-all.

The C-index of the SVM model in the estimation of progression was 0.652. Comparison of the C-indexes and AUC values of the multivariate CPH, CLIPI, and SVM models in the estimation of progression are shown in Table 7.

#### 3.2.3. Complete Response

**Figure 6:**
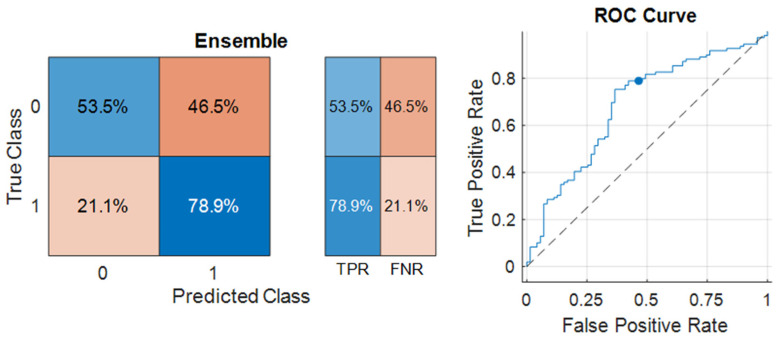
Confusion matrix and ROC Curve of the best-performing model in the estimation of complete response.

The most successful model in predicting the complete response was the Ensemble model, with 68.89% accuracy and an F1 Score of 75.44% achieved (given in Table 8). The sensitivity (TPR) of the model was 78.9%, and the specificity (TNR) was 53.5% (Figure 6). The AUC of the model was 0.68226. The ROC Curve for the Ensemble model was also illustrated in Figure 3. The optimum hyperparameters were also obtained by the Bayesian optimization for the Ensemble model. Accordingly, the optimum ensemble method was determined as GentleBoost, and the optimum value for the maximum number of splits was 14, the number of learners was 15, and the learning rate was 0.001.

The C-index of the Ensemble model in the estimation of complete response was 0.662. Comparison of the C-indexes and AUC values of the multivariate CPH and Ensemble models in the estimation of complete response are shown in Table 9.

#### 3.2.4. Relapse

**Figure 7:**
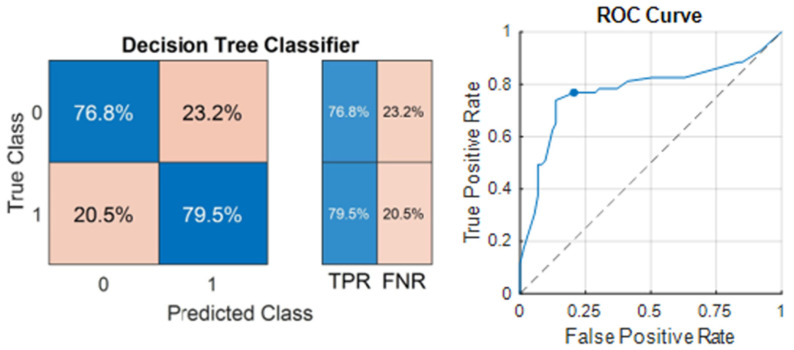
Confusion matrix and ROC Curve of the best-performing model in the estimation of relapse.

The most successful model in predicting relapse was the Tree model, and 78.17% accuracy was achieved, with an F1 Score of 78.91 (given in Table 10). The sensitivity of the model was 79.5%, and the specificity was 76.8% (Figure 7). The AUC of the model was 0.77546. The ROC Curve for the decision tree classifier is also illustrated in Figure 4. The optimum hyperparameters were also obtained by the Bayesian optimization for the Tree model. Accordingly, the optimum maximum number of splits was determined as eight, and the split criterion was Gini’s diversity index.

The C-index of the decision tree classifier in the estimation of relapse was 0.782. Comparisons of the C-indexes and AUC values of the multivariate CPH and decision tree models in the estimation of relapse are shown in Table 11.

## 4. Discussion

The significance of artificial intelligence in the practice of dermatology is increasing. Existing academic research primarily focuses on diagnosing diseases using artificial intelligence [17,18]. Nevertheless, the literature does contain studies on the use of ML in predicting the prognosis of malignancies [35]. Cheraghlou et al. employed the modified classification and regression tree method, an ML technique, to estimate survival rates in patients with Merkel cell carcinoma. Patient demographic and clinical data were analyzed for risk stratification, and the algorithm demonstrated a noteworthy accuracy in survival prediction [36]. In another study, Damiani and colleagues evaluated the data of squamous cell carcinoma patients using an artificial neural network (ANN)-based algorithm to identify patterns of patients with higher risk of postradiotherapy recurrence [37]. In our research, we evaluated the performances of several ML algorithms in predicting the initial treatment response, relapse, and progression in early-stage MF patients by using patient data.

The machine learning (ML) methods employed in this study are generally more adept at multi-class prediction and handling intricate decision boundaries when compared to conventional statistical techniques, such as Cox proportional hazards (CPH) models and multivariate regression analyses. Traditional statistical methods often encounter challenges with nonlinear relationships and datasets featuring a high dimensionality [38]. In contrast to ML techniques, which exhibit high flexibility and operate without predefined assumptions, traditional statistical methodologies rely on strict assumptions necessitating an explicit specification of the relationship between independent and dependent variables (e.g., interaction terms, polynomial terms). Moreover, traditional statistical models are limited by the researcher’s capacity to formulate hypotheses concerning these connections [39].

ML models excel at capturing nonlinear associations among variables, accommodating high-dimensional data, and performing effectively with smaller sample sizes [39]. Numerous studies have revealed that ML models, particularly in tasks related to disease progression and prediction, frequently outperform traditional statistical methods regarding predictive accuracy and adaptability across diverse clinical scenarios [40,41]. In accordance with the existing literature, our study illustrated that ML methods surpassed CPH models regarding the prediction of progression, relapse, and complete response, as indicated by superior C-index and AUC outcomes.

Numerous studies have been conducted previously on the prognosis of mycosis fungoides. CLIPI prognostic index was devised using overall survival (OS) and progression-free survival (PFS) as key endpoints. The index identifies distinct criteria for patients in both the early and advanced stages of MF and demonstrates a strong correlation to overall and progression-free survival rates in both patient groups [9]. However, subsequent research did not validate the association between the CLIPI index and prognosis [10,11]. The study’s definition of PFS—the time from diagnosis to stage progression (excluding changes from T1a to T1b or T2a to T2b) or disease-specific death—differed from the commonly accepted definitions set by the ISCL, including those used in our study [13]. The definition determined by the ISCL and applied in our study is more detail-oriented and, thus, may be more useful and decisive in early-stage MF. Additionally, using OS as an endpoint may introduce bias, especially in early-stage MF, due to short follow-up periods in most studies. Furthermore, likely due to these aforementioned pitfalls, CLIPI could not be validated in other cohorts with early-stage MF [11]. Similarly, the CLIPI index was inferior to the SVM model in predicting progression in our cohort (C-index: 0.652 for SVM vs. 0.278 for CLIPI; AUC: 0.73 for SVM vs. 0.65 for CLIPI). Nevertheless, upon comparing significant factors, in the CLIPI study, male gender, age over 60 years, plaque lesion, folliculotropism, and lymphadenopathy were statistically linked with PFS in the early stage [9]. In our study, factors evaluated using ML algorithms were prioritized based on their significance in predicting the risk of progression. Gender and folliculotropism emerged as the most important factors for progression, similar to the CLIPI study. However, age, lesion type, and lymphadenopathy were not among the most important factors in progression.

Male gender, advanced age, stage increase, plaque lesions, raised lactate dehydrogenase and ß2 microglobulin levels, blood eosinophilia, folliculotropism, and large-cell transformation in pathology have been defined as poor prognostic factors; conversely, characteristics like patch lesions, hypopigmentation, poikiloderma, lymphomatoid papulosis, and CD8 positivity in immunohistochemical analyses are defined as favorable prognostic factors [4,7,9,42,43,44]. Despite the isolated prognostic insights these factors provide, there is a lack of research that cumulatively integrates these factors to predict the prognosis of MF. Our study fills this gap by recording comprehensive patient data, including demographic, clinical, laboratory, and pathological information, and subsequently training ML algorithms for predictive purposes. The most successful predictions for progression were obtained with the SVM algorithm, with an accuracy of 75%. The algorithm demonstrated a sensitivity of 40.7% and a specificity of 89.7%. The most important features in predicting progression in the current study were folliculotopism, lymphomatoid papulosis, gender, LDH, and β-2 microglobulin levels consistent with the literature. Remarkably, the LDH value proved to be significant in predicting progression in both univariate and multivariate CPH models, as well as in ML analyses. The significance of skin-directed treatment, shown to be effective in the univariate analysis and machine learning, may be attributed to its preference among patients believed to be at low risk of progression. As the follow-up duration extends, the detection of progression and recurrence becomes more straightforward. The noteworthy impact of the follow-up period in our research emphasizes the necessity for vigilant monitoring of patients at risk of progression and recurrence.

The therapeutic objective for early-stage MF involves ensuring total lesion regression and preventing relapses. Numerous clinical, laboratory, and pathological features linked with treatment response and relapse have been described in the literature [12,45,46,47,48]. Features such as folliculotropism, hyperpigmented morphology, lymphomatoid papulosis, LDH level, eosinophilia, disease stage, and skin-directed treatment were identified as important features influencing complete response, as in progression in our research. Furthermore, β-2 microglobulin levels emerged as a crucial feature in both the CPH multivariate analysis and machine learning models. Patients with these features may exhibit incomplete responses to initial treatment. In contrast, a complete response is more probable in cases of lymphomatoid papulosis and the hyperpigmented morphological type, which are linked to an indolent disease course [49]. Regarding recurrence, the primary characteristics included the duration of follow-up, age at onset of lesions, age at diagnosis, gender, and time to diagnosis. Patients experiencing recurrence tended to have a longer follow-up period and time to diagnosis and a shorter duration between the onset of lesions and diagnosis age, with a predominance of male gender. The Tree algorithm was the most successful algorithm in predicting relapse, with 78.17% accuracy, 79.5% sensitivity, and 78.8% specificity. The Ensemble algorithm demonstrated the highest level of accuracy in predicting treatment response, although its accuracy rates were somewhat lower compared to predictions of progression and relapse, with an accuracy of 68.89% (showing high sensitivity at 78.9% and lower specificity at 53.5%). Despite these findings, the ML algorithms’ success rates could be enhanced by training with larger sample groups. These results suggested that such algorithms could be leveraged to predict the course of MF using patient data and assist medical professionals in planning patient management.

In our research, while predicting progression, relapse, and complete response, several discrepancies were observed between the variables deemed significant by the CPH model and those identified as important by the machine learning methods. These differences may be explained by several factors beyond those already discussed. First, the heightened influence of the top five features in the machine learning assessment, compared to other factors that, while still important, rank lower in significance, could be crucial in this context [50]. Second, the limitations of incorporating an insufficient number of variables in the CPH model hinder its ability to comprehensively evaluate all parameters [51]. Additionally, the abundance of predictors, combined with the relatively modest sample size of our dataset, may also contribute to these discrepancies [52].

There are certain limitations to our study. First, it can be considered that the retrospective design of our study is a limitation as it may cause patient selection bias. Secondly, since it is a single-center study, the sample size is relatively restricted.

## 5. Conclusions

While our study demonstrates the potential of ML models such as decision trees, discriminant classifiers, logistic regression, Naive Bayes classifiers, SVMs, KNNs, Ensemble methods, and shallow neural networks in predicting treatment response, progression, and risk of relapse in early-stage MF patients, several limitations should be acknowledged. One key limitation is that these models often require careful feature selection and tuning to perform well in clinical datasets with limited sample sizes, such as ours. Furthermore, their generalizability may be restricted due to the heterogeneity of data across different cohorts. Although deep learning models were not included in this study due to their need for larger datasets to achieve optimal performance, they represent a promising avenue for future research as more data become available with multi-center collaborations. Potential applications of ML in MF prognosis extend beyond the prediction of progression and relapse, including personalized treatment planning and identifying novel prognostic biomarkers. However, challenges such as the interpretability of complex models, the risk of overfitting, and the need for external validation in diverse patient populations must be addressed to ensure clinical utility and widespread adoption.

## Figures and Tables

**Table 1:** Demographic and clinical characteristics of patients.

		Number of Patients (%)
Gender	Female	94 (50.8)
	Male	91 (49.2)
Stage at Diagnosis	IA	76 (41.0)
	IB	73 (39.5)
	IIA	36 (19.5)
T Stage at Diagnosis	T1	88 (47.6)
	T2	97 (52.4)
N Stage at Diagnosis	N0	82 (44.3)
	Nx	67 (36.2)
	N1	36 (19.5)
B Stage at Diagnosis	B0	180 (97.3)
	B1	5 (2.7)
Patch/Plaque	Patch	79 (42.7)
	Patch + Plaque	106 (57.3)
Morphological Findings	Erythematous	120 (64.8)
	Hyperpigmented	35 (18.9)
	Poikiloderma	13 (7.0)
	Folliculotropism	9 (4.8)
	PPD-like	7 (3.8)
	Hypopigmented	3 (1.6)
	GSS	1 (0.5)
	Ichthyosiform	1 (0.5)
Pruritus	No	77 (41.6)
	Yes	108 (58.4)
LDH Levels	Normal	170 (91.9)
	High	15 (8.1)
β-2 Microglobulin Levels	Normal	121 (65.4)
	High	64 (34.6)
Eosinophilia	No	174 (94.0)
	Yes	11 (6.0)
Histopathological Findings	Classic	119 (64.3)
	Folliculotropism	28 (15.1)
	LCT	9 (4.8)
	PPD-like	5 (2.7)
	GSS	1 (0.5)
	Syringotropic	1 (0.5)
	Granuloma Annulare-like	1 (0.5)
Immunohistochemical Findings	CD4 (+) CD8 (−)	153 (82.7)
	CD4 (−) CD8 (+)	28 (15.1)
	CD4 (−) CD8 (−)	2 (1.0)
	CD4 (+) CD8 (+)	2 (1.0)
	CD30 (+)	4 (2.1)
Initial Treatment Type	Skin-directed	149 (80.0)
	Systemic	6 (3.3)
	Combined	30 (16.7)
Initial Treatment	Topical Corticosteroid	6 (3.3)
	Bexarotene Gel	1 (0.5)
	nbUVB	65 (35.1)
	PUVA	77 (41.6)
	PUVA + Bexarotene Gel	1 (0.5)
	Methotrexate (MTX)	2 (1.0)
	Acitretin	3 (1.6)
	Interferon (IFN)	1 (0.5)
	nbUVB + Acitretin	1 (0.5)
	nbUVB + IFN	1 (0.5)
	PUVA + Acitretin	2 (1.0)
	PUVA + IFN	24 (12.9)
	PUVA + MTX	1 (0.5)
	ECP + IFN	1 (0.5)
		Period (Mean)
Age at Diagnosis (Years)		50.8
Age of Onset of Lesions (Years)		45.9
Follow-up Time (Years)		5.6
Time from Appearance to Diagnosis (Months)		56.3

Pigmented Purpuric Dermatosis (PPD); Granulomatous Slack Skin (GSS); lactate dehydrogenase (LDH); Large Cell Transformation (LCT); Narrowband Ultraviolet B (nbUVB); Psoralen Ultraviolet A (PUVA); Methotrexate (MTX); Interferon (IFN); Extracorporeal Photopheresis (ECP).

**Table 2:** Patient characteristics according to progression, complete response, and relapse.

		Progression *n* (%)		Complete Response *n* (%)		Relapse *n* (%)	
		No	Yes	No	Yes	No	Yes
Gender	Female	74 (56.5)	20 (37.0)	36 (50.7)	58 (50.9)	25 (55.6)	33 (47.8)
	Male	57 (43.5)	34 (63.0)	35 (49.3)	56 (49.1)	20 (44.4)	36 (52.2)
Stage at Diagnosis	IA	54 (41.2)	22 (40.7)	22 (31)	54 (47.4)	24 (53.3)	30 (43.5)
	IB	53 (40.4)	20 (37.0)	28 (39.4)	45 (39.5)	16 (35.6)	29 (42.0)
	IIA	24 (18.3)	12 (22.3)	21 (29.6)	15 (13.1)	5 (11.1)	10 (14.5)
T Stage at Diagnosis	T1	59 (45.0)	29 (53.7)	31 (43.7)	57 (50.0)	26 (57.8)	31 (44.9)
	T2	72 (55.0)	25 (46.3)	40 (56.3)	57 (50.0)	19 (42.2)	38 (55.1)
N Stage at Diagnosis	N0	67 (51.1)	15 (27.8)	24 (33.8)	58 (50.9)	24 (53.3)	34 (49.3)
	Nx	40 (30.5)	27 (50.0)	26 (36.6)	41 (36.0)	16 (35.6)	25 (36.2)
	N1	24 (18.4)	12 (22.2)	21 (29.6)	15 (13.1)	5 (11.1)	10 (14.5)
B Stage at Diagnosis	B0	128 (97.7)	52 (96.3)	68 (95.8)	112 (98.3)	45 (100)	67 (95.9)
	B1	3 (2.3)	2 (3.7)	3 (4.2)	2 (1.7)	0 (0)	2 (4.1)
Patch/Plaque	Patch	59 (45.0)	20 (37.0)	26 (36.6)	53 (46.5)	23 (51.1)	30 (43.5)
	Patch + Plaque	72 (55.0)	34 (63.0)	45 (63.4)	61 (53.5)	22 (48.9)	39 (56.5)
Morphological Findings	Erythematous	88 (67.1)	32 (59.2)	48 (67.6)	72 (63.2)	27 (60.0)	45 (63)
	Hyperpigmented	27 (20.6)	8 (14.8)	13 (18.3)	22 (19.3)	8 (17.8)	14 (16.4)
	Poikiloderma	6 (4.5)	7 (13.0)	6 (8.5)	7 (6.1)	2 (4.4)	5 (9.6)
	Folliculotropism	5 (3.8)	4 (7.4)	5 (5.6)	4 (4.4)	1 (2.2)	3 (5.5)
	PPD-like	6 (4.5)	1 (1.9)	1 (1.4)	6 (5.3)	4 (8.9)	2 (4.1)
	Hypopigmented	2 (1.5)	1 (1.9)	0 (0)	3 (2.6)	3 (6.7)	0
	GSS	0 (0)	1 (1.9)	1 (1.4)	0 (0)	-	-
	Ichthyosiform	0 (0)	1 (1.9)	1 (1.4)	0 (0)	-	-
Pruritus	No	58 (44.3)	19 (35.2)	28 (39.4)	49 (43.0)	20 (44.4)	29 (42.0)
	Yes	73 (55.7)	35 (64.8)	43 (60.6)	65 (57.0)	25 (55.6)	40 (58.0)
LDH Levels	Normal	126 (96.2)	44 (81.5)	61 (86.0)	109 (95.6)	42 (93.3)	67 (97.1)
	High	5 (3.8)	10 (18.5)	10 (14.0)	5 (4.4)	3 (6.7)	2 (2.9)
β-2 Microglobulin Levels	Normal	94 (71.8)	27 (50.0)	36 (50.7)	85 (74.6)	35 (77.8)	50 (72.5)
	High	37 (28.2)	27 (50.0)	35 (49.3)	29 (25.4)	10 (22.2)	19 (27.5)
Eosinophilia	No	124 (94.7)	50 (92.6)	64 (90.1)	110 (96.5)	44 (97.8)	66 (95.7)
	Yes	7 (5.3)	4 (7.4)	7 (9.9)	4 (3.5)	1 (2.2)	3 (4.3)
Histopathological Findings	Classic	90 (68.7)	29 (53.7)	39 (55.7)	80 (85.1)	30 (66.7)	50 (72.5)
	Folliculotropism	15 (11.5)	13 (24.0)	18 (25.7)	10 (10.6)	4 (8.9)	6 (8.7)
	LCT	6 (4.6)	3 (5.5)	9 (12.9)	0 (0)	-	-
	PPD-like	4 (3.1)	1 (1.9)	2 (2.9)	3 (3.2)	1 (2.2)	2 (2.9)
	GSS	0 (0)	1 (1.9)	1 (1.4)	0 (0)	-	-
	Syringotropic	1 (0.7)	0 (0)	0 (0)	1 (1.1)	0 (0)	1 (1.4)
	Granuloma Annulare-like	1 (0.7)	0 (0)	1 (1.4)	0 (0)	-	-
Immunohistochemical Findings	CD4 (+) CD8 (−)	113 (86.3)	40 (74.0)	61 (81.3)	92 (80.9)	33 (73.3)	59 (85.5)
	CD4 (−) CD8 (+)	22 (16.8)	6 (11.1)	8 (10.7)	20 (17.5)	11 (24.4)	9 (13.0)
	CD4 (−) CD8 (−)	0 (0)	2 (3.7)	1 (1.3)	1 (0.8)	0 (0)	1 (1.4)
	CD4 (+) CD8 (+)	1 (0.7)	1 (1.9)	1 (1.3)	1 (0.8)	1 (2.2)	0 (0)
	CD30 (+)	1 (0.7)	3 (5.5)	4 (5.4)	0 (0)	-	-
Initial Treatment Type	Skin-directed	111 (84.7)	38 (70.3)	52 (73.2)	97 (85.1)	39 (86.7)	58 (84.1)
	Systemic	1 (0.7)	5 (9.3)	4 (5.6)	2 (1.7)	0 (0)	2 (2.9)
	Combined	19 (14.5)	11 (20.4)	15 (21.1)	15 (13.2)	3 (13.3)	12 (13.0)
Initial Treatment	Topical Corticosteroid	4 (3.1)	2 (3.7)	3 (4.2)	3 (2.6)	0 (0)	3 (5.5)
	Bexarotene Gel	1 (0.7)	0 (0)	0 (0)	1 (0.8)	1 (2.2)	0 (0)
	nbUVB	51 (38.9)	14 (25.9)	22 (31.0)	43 (37.7)	18 (40.0)	25 (36.2)
	PUVA	55 (41.9)	21 (38.8)	27 (38.0)	49 (43.0)	18 (40.0)	31 (44.9)
	PUVA + Bexarotene Gel	0 (0)	1 (1.9)	0 (0)	1 (0.8)	0 (0)	1 (1.4)
	Methotrexate (MTX)	1 (0.7)	1 (1.9)	1 (1.4)	1 (0.8)	1 (2.2)	0 (0)
	Acitretin	1 (0.7)	2 (3.7)	2 (2.8)	1 (0.8)	1 (2.2)	0 (0)
	Interferon (IFN)	0 (0.7)	1 (1.9)	1 (1.4)	0 (0)	-	-
	nbUVB + Acitretin	1 (0.7)	0 (0)	0 (0)	1 (0.8)	1 (2.2)	0 (0)
	nbUVB + IFN	0 (0)	1 (1.9)	1 (1.4)	0 (0)	-	-
	PUVA + Acitretin	2 (1.5)	0 (0)	1 (1.4)	1 (0.8)	0 (0)	1 (1.4)
	PUVA + IFN	13 (9.9)	11 (20.4)	13(18.3)	11 (9.6)	3 (6.7)	8 (11.6)
	PUVA + MTX	1 (0.7)	0 (0)	0 (0)	1 (0.8)	1 (2.2)	0 (0)
	ECP + IFN	1 (0.7)	0 (0)	0 (0)	1 (0.8)	1 (2.2)	0 (0)
Age at Diagnosis		50.6	51.2	52.4	49.8	53	47.7
Age of Onset of Lesions (Years)		46.3	44.9	47.6	44.8	48.3	42.5
Follow-up Time (Years)		5.1	6.8	5.7	5.5	3.0	7.1
Time from Appearance to Diagnosis (Months)		52.1	66.5	57.4	55.6	42.5	64.1

Pigmented Purpuric Dermatosis (PPD); Granulomatous Slack Skin (GSS); lactate dehydrogenase (LDH); Large Cell Transformation (LCT); Narrowband Ultraviolet B (nbUVB); Psoralen Ultraviolet A (PUVA); Methotrexate (MTX); Interferon (IFN); Extracorporeal Photopheresis.

**Table 3:** Univariate and multivariate Cox proportional hazards model results for progression.

Progression						
	Univariate Analysis			Multivariate Analysis		
	*p*-Value	Hazard Ratio	%95 CI	*p*-Value	Hazard Ratio	%95 CI
Age at diagnosis	0.029 *	1.023	1.002–1.043	0.261	1.013	0.990–1.036
Age of Onset of Lesions (Years)	0.214	1.012	0.993–1.031	-	-	-
Time from Appearance to Diagnosis (Months)	0.064	1.003	1.000–1.005	-	-	-
Gender (Male)	0.715	1.111	0.630–1.962	-	-	-
Patch/Patch + Plaque (Patch + Plaque)	0.958	1.015	0.580–1.777	-	-	-
Morphological Findings						
Hyperpigmentation (Yes)	0.092	0.522	0.245–1.112	-	-	-
Erythema (Yes)	0.787	1.079	0.620–1.878	-	-	-
Pruritus (Yes)	0.035 *	1.906	1.046–3.473	0.153	1.624	0.835–3.155
Histopathological Findings						
Classic (Yes)	0.015 *	0.498	0.051–0.873	0.022 *	0.477	0.258–0.901
Folliculotropism (Yes)	0.085	2.502	0.882–7.096	-	-	-
CD8 (+) (Yes)	0.920	0.960	0.431–2.139	-	-	-
Stage at Diagnosis						
IB	0.890	0.957	0.516–1.776	-	-	-
IIA	0.667	0.856	0.421–1.740	-	-	-
T Stage at Diagnosis (T2)	0.319	0.756	0.437–1.310	-	-	-
N Stage at Diagnosis						
Nx	0.009 *	2.404	1.250–4.623	0.905	0.951	0.417–5.555
N1	0.272	1.520	0.720–3.210	-	-	-
β-2 Microglobulin (High)	0.238	1.388	0.805–2.391	-	-	-
LDH (High)	0.038 *	2.107	1.041–4.267	0.004 *	3.424	1.475–7.947
Eosinophilia (Yes)	0.112	2.350	0.820–6.735	-	-	-
Initial Treatment Type						
Skin-directed	0.005 *	0.415	0.210–0.770	0.581	0.809	0.382–1.716
Combined	0.170	1.615	0.814–3.205	-	-	-
Initial Treatment						
NB-UVB	0.096	1.694	0.911–3.149	-	-	-
PUVA	0.022 *	0.524	0.112–0.911	0.313	0.722	0.384–1.359
PUVA + IFN	0.757	1.146	0.484–2.710	-	-	-

* *p* < 0.05 significant relationship. *p* > 0.05 no significant relationship; CI: confidence interval; LDH: lactate dehydrogenase; NB-UVB: Narrowband Ultraviolet B; PUVA: Psoralen + Ultraviolet A; IFN: Interferon.

**Table 4:** Univariate and multivariate Cox proportional hazards model results for complete response.

Complete Response						
	Univariate Analysis			Multivariate Analysis		
	*p*-Value	Hazard Ratio	%95 CI	*p*-Value	Hazard Ratio	%95 CI
Age at Diagnosis	0.043 *	1.014	1.001–1.028	0.065	1.015	0.999–1.030
Age of Onset of Lesions (Years)	0.140	1.010	0.997–1.022	-	-	-
Time to Diagnosis (Months)	0.314	1.001	0.999–1.003	-	-	-
Gender (Male)	0.030 *	0.660	0.250–0.960	0.028 *	0.638	0.150–0.952
Patch/Patch + Plaque (Patch + Plaque)	0.056	0.696	0.480–1.009	-	-	-
Morphological Findings						
Hyperpigmentation (Yes)	0.377	0.815	0.519–1.282	-	-	-
Erythema (Yes)	0.069	1.444	0.972–2.145	-	-	-
Pruritus (Yes)	0.369	1.191	0.813–1.743	-	-	-
Histopathological Findings						
Classic (Yes)	0.770	1.062	0.708–1.593	-	-	-
Folliculotropism (Yes)	0.870	1.078	0.437–2.659	-	-	-
CD8 (+) (Yes)	0.360	1.254	0.772–2.037	-	-	-
Stage at Diagnosis						
IB	0.718	0.930	0.627–1.380	0.813	1.050	0.703–1.567
IIA	0.003 *	0.402	0.192–0.737	0.863	1.194	0.159–8.960
T Stage at Diagnosis (T2)	0.552	0.894	0.618–1.294	-	-	-
N Stage at Diagnosis						
Nx	0.059	0.382	0.141–1.039	-	-	-
N1	0.680	0.809	0.296–2.214	-	-	-
β-2 Microglobulin (High)	0.003 *	0.535	0.110–0.812	0.012 *	0.567	0.254–0.883
LDH (High)	0.059	0.382	0.141–1.039	-	-	-
Eosinophilia (Yes)	0.680	0.809	0.296–2.213	-	-	-
Initial Treatment Type						
Skin-directed	0.571	1.155	0.702–1.900	-	-	-
Combined	0.850	0.952	0.573–1.583	-	-	-
Initial Treatment						
NB-UVB	0.001 *	0.538	0.250–0.785	0.769	0.922	0.536–1.587
PUVA	0.001 *	1.990	1.338–2.961	0.018 *	1.870	1.114–3.139
PUVA + IFN	0.118	5.114	0.663–39.463	-	-	-

* *p* < 0.05 significant relationship. *p* > 0.05 no significant relationship; CI: confidence interval; LDH: lactate dehydrogenase; NB-UVB: Narrowband Ultraviolet B; PUVA: Psoralen + Ultraviolet A; IFN: Interferon.

**Table 5:** Univariate and multivariate Cox proportional hazards model results for relapse.

Relapse						
	Univariate Analysis			Multivariate Analysis		
	*p*-Value	Hazard Ratio	%95 CI	*p*-Value	Hazard Ratio	%95 CI
Age at Diagnosis	0.585	1.005	0.987–1.023	-	-	-
Age of Onset of Lesions (Years)	0.615	0.996	0.980–1.012	-	-	-
Time to Diagnosis (Months)	0.032 *	1.002	1.001–1.005	0.037 *	1.003	1.001–1.005
Gender (Male)	0.474	0.837	0.515–1.361	-	-	-
Patch/Patch + Plaque (Patch + Plaque)	0.177	0.709	0.431–1.167	-	-	-
Morphological Findings						
Hyperpigmentation (Yes)	0.211	0.691	0.387–1.233	-	-	-
Erythema (Yes)	0.909	1.030	0.625–1.695	-	-	-
Pruritus (Yes = 1)	0.515	1.176	0.722–1.914	-	-	-
Histopathological Findings						
Classic	0.270	0.753	0.455–1.246	-	-	-
Folliculotropism	0.579	1.392	0.433–4.477	-	-	-
CD8 (+)	0.895	0.957	0.501–1.830	-	-	-
Stage at Diagnosis						
IB	0.281	0.786	0.457–1.351	-	-	-
IIA	0.518	0.892	0.460–1.733	-	-	-
T Stage at Diagnosis (T2)	0.815	0.944	0.583–1.529	-	-	-
N Stage at Diagnosis						
Nx	0.560	0.827	0.483–1.416	-	-	-
N1	0.492	0.815	0.412–1.613	-	-	-
β-2 Microglobulin (High)	0.679	0.899	0.541–1.492	-	-	-
LDH (High)	0.210	2.475	0.599–10.226	-	-	-
Eosinophilia (Yes)	0.869	0.887	0.214–3.674	-	-	-
Initial Treatment Type						
Skin-directed	0.787	1.098	0.557–2.165	-	-	-
Combined	0.940	0.974	0.494–1.920	-	-	-
Initial Treatment						
NB-UVB	0.001 *	2.860	1.693–4.831	0.134	1.672	0.853–3.277
PUVA	0.000 *	0.375	0.101–0.628	0.034 *	0.485	0.210–0.947
PUVA + IFN	0.327	1.693	0.729–2.578	-	-	-

* *p* < 0.05 significant relationship. *p* > 0.05 no significant relationship; CI: confidence interval; LDH: lactate dehydrogenase; NB-UVB: Narrowband Ultraviolet B; PUVA: Psoralen + Ultraviolet A; IFN: Interferon.

**Table 6:** Ten-fold cross-validation results of the ML models in the estimation of progression.

Model Type	Accuracy %	F1 Score %
**SVM**	**75.00**	**49.44**
Discriminant	73.89	48.35
KNN	72.78	24.62
BGLM Logistic Regression	72.22	41.86
Efficient Logistic Regression	71.67	37.04
Efficient Linear SVM	71.67	38.55
Ensemble	71.11	23.53
Tree	70.56	18.46
Neural Network	68.89	46.15
Naive Bayes	65.56	41.51

The most successful model is marked in bold.

**Table 7:** The C-indexes and AUC values of the best-performing machine learning model, CILIPI, and the CPH model in the estimation of progression.

	C-Index	AUC
CPH	0.501	0.451
SVM	0.652	0.73927
CLIPI	0.278	0.655

**Table 8:** Ten-fold cross-validation results of the ML models in the estimation of complete response.

Model Type	Accuracy %	F1 Score %
**Ensemble**	**68.89**	**75.44**
Neural Network	66.11	74.26
SVM	66.11	74.69
KNN	65.56	72.81
Naive Bayes	65.00	75.1
BGLM Logistic Regression	65.00	72.96
Efficient Logistic Regression	63.33	72.5
Discriminant	61.11	69.03
Efficient Linear SVM	61.11	71.77
Tree	60.00	72.73

The most successful model is marked in bold.

**Table 9:** The C-indexes and AUC values of the best-performing machine learning model and the CPH model in the estimation of complete response.

	C-Index	AUC
CPH	0.543	0.36
Ensemble	0.662	0.68226

**Table 10:** Ten-fold cross-validation results of the ML models in the estimation of relapse.

Model Type	Accuracy %	F1 Score %
**Tree**	**78.17**	**78.91**
Ensemble	77.46	78.08
SVM	70.42	71.62
Discriminant	62.68	62.94
KNN	62.68	60.74
Efficient Linear SVM	62.68	62.94
Naive Bayes	60.56	67.44
Neural Network	59.15	57.35
Efficient Logistic Regression	57.75	58.9
BGLM Logistic Regression	57.04	57.93

The most successful model is marked in bold.

**Table 11:** The C-indexes and AUC values of the best-performing machine learning model and the CPH model in the estimation of relapse.

	C-Index	AUC
CPH	0.505	0.489
Decision Tree	0.782	0.77546

## Data Availability

The data featured in this work can be obtained with a special request from the corresponding author.
